# Fair at the New York State Lunatic Asylum

**Published:** 1844-02

**Authors:** 


*Fair at the New York State Lunatic Asylum.*—A fair, as we learn by the Utica papers, was held at the Lunatic Asylum, in that city, on the 18th.

"The table was spread in the lower hall of the eastern wing, and extended about two-thirds of its entire length, which is some 250 feet. Most of the patients' rooms on that floor were thrown open, and all the inmates of the Asylum who were in sufficient health, were permitted to mingle with the gay and busy throng from the outer world which crowded the hall. Among those actively engaged in the management of the fair, the distinguished female philanthropist, Miss Dix, who, in the prosecution of her benevolent labors, has been for some weeks a visitor at the Asylum, attracted much attention.

"This is the first thing of the sort, we presume, ever attempted within the walls of a lunatic asylum. We trust it may prove the source of as much benefit to the inmates as it was of pleasure to the visitors. The public are much indebted to the able superintendent, Dr. Brigham, and the other officers of the institution, for arrangements and attentions which made the whole affair pass off in the most agreeable manner.

"We learn that a fund of about $200 has been realized from the fair, which will be expended for books, musical instruments, &c. It was a source of much amusement to the patients, and its beneficial effects are strikingly apparent with some of them. We are further informed that some dozen of the patients are writing accounts of the fair, but it is not known whether their productions are intended for 'the public eye.'"

